# Endoscopic-Assisted Hematoma Evacuation Through a Small Craniotomy for Acute Subdural Hematoma Presenting With “Talk and Deteriorate”: A Case Report

**DOI:** 10.7759/cureus.100531

**Published:** 2025-12-31

**Authors:** Ryo Matsuzaki, Yuki Sakaeyama, Sayaka Terazono, Shuhei Kubota, Nobuo Sugo

**Affiliations:** 1 Neurosurgery, Toho University, Tokyo, JPN

**Keywords:** acute subdural hematoma, craniotomy, endoscopic hematoma evacuation, rigid endoscope, surgical invasion

## Abstract

Craniotomy has traditionally been the standard procedure for acute subdural hematoma (ASDH). As ASDH occurs predominantly in the elderly, its incidence is expected to increase in aging societies. In elderly patients with comorbidities, craniotomy may not always be feasible due to its invasiveness and long operative time. Endoscopic-assisted hematoma evacuation can potentially reduce operative time and surgical invasiveness. Here, we report a case treated with endoscopic-assisted hematoma evacuation and discuss its indications and technical considerations.

## Introduction

Acute subdural hematoma (ASDH) is an accumulation of blood in the subdural space, most commonly after traumatic brain injury (TBI), and can be rapidly fatal because it increases intracranial pressure and may cause midline shift and brain herniation [1]. Clinically, patients may present with headache, vomiting, focal neurological deficits, or a decline in consciousness, including a “talk-and-deteriorate” course. ASDH is traditionally treated by craniotomy for hematoma evacuation and hemostasis [1]. Standard open surgical strategies include craniotomy, decompressive craniectomy, and, in selected urgent cases, trephination or craniostomy. The choice of procedure is individualized based on neurological status, radiological findings, and the degree of intraoperative brain swelling [1]. Importantly, ASDH is not uniformly managed with a large decompressive craniectomy; rather, the extent of bone removal is tailored to the anticipated swelling and the need for decompression and hemostasis [1].

Despite advances in trauma systems and surgical techniques, outcomes in severe ASDH remain unfavorable, with reported mortality remaining high, particularly in patients presenting with poor neurological status [1]. ASDH frequently occurs in elderly patients, and its incidence is expected to rise with population aging and widespread antithrombotic use [1]. While craniotomy remains indicated for large hematomas or those with significant swelling, reports of endoscopic-assisted evacuation remain limited [1,2]. Compared with conventional craniotomy using a wide operative corridor, endoscopic-assisted hematoma evacuation aims to achieve adequate clot removal and bleeding control through a smaller exposure, potentially reducing surgical stress in selected patients [3-5]. It has been performed under local or general anesthesia, depending on patient status and intraoperative factors [3-5].

However, endoscopic-assisted evacuation is not uniformly quicker or technically easier, particularly in acute ASDH with brain swelling and high intracranial tension, where endoscopic manipulation may be challenging and time-consuming. Although its indication is limited in cases with marked brain swelling, these constraints underscore the importance of careful patient selection, and appropriate case selection may allow for a safe and minimally invasive approach [6].

## Case presentation

**Figure 1:**
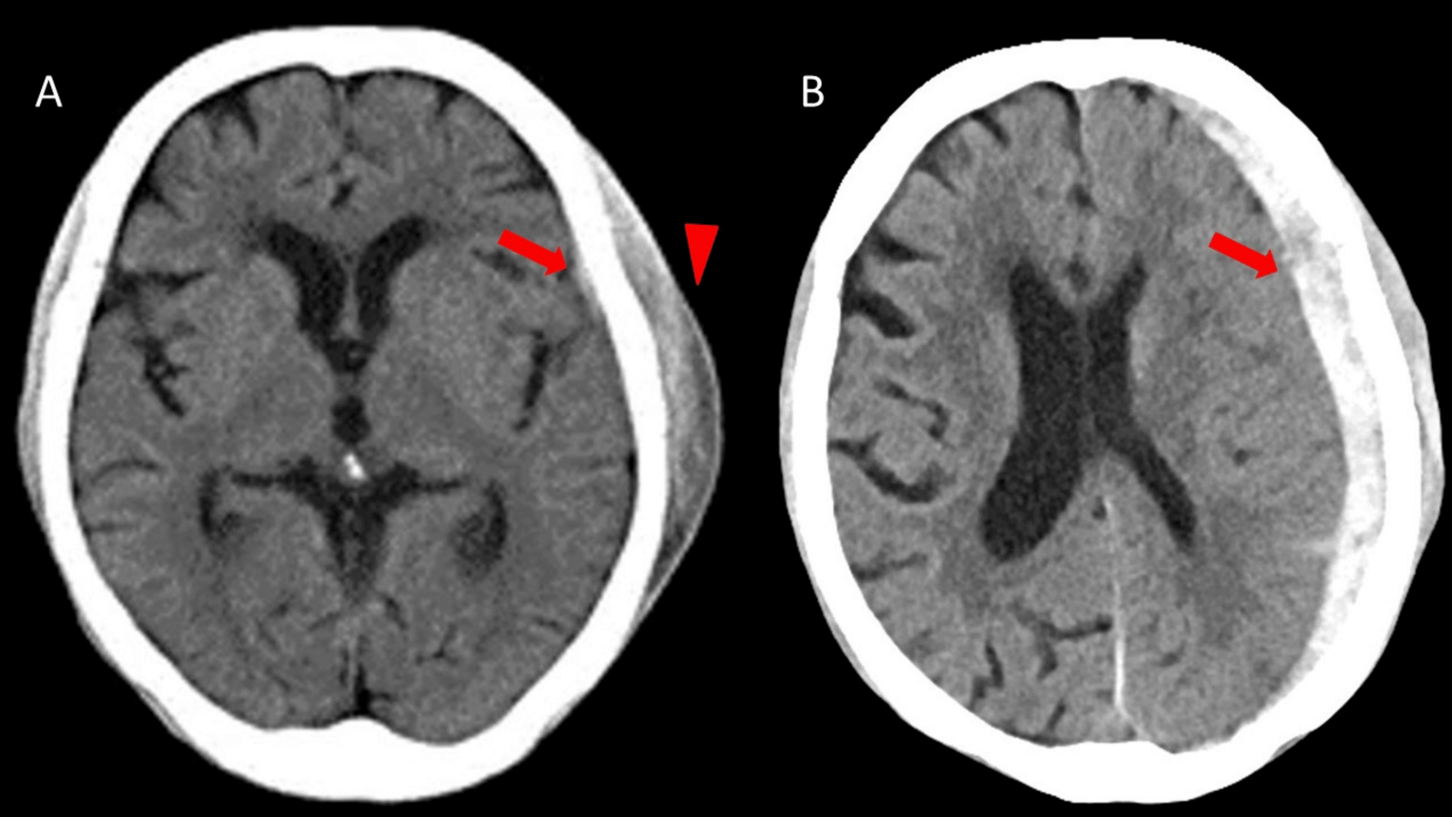
(A) Head CT obtained at another hospital on the day of injury showing a thin acute subdural hematoma (arrow) along the left cerebral convexity and an overlying scalp (subcutaneous) hematoma (arrowhead). (B) Head CT obtained at our hospital seven days after the injury showing enlargement of the acute subdural hematoma (arrow) with increased mass effect on the brain (midline shift).

A 76-year-old woman sustained a head injury from a fall (Day 0). Initial head CT at another hospital demonstrated a thin left ASDH measuring 3 mm in thickness (Figure 1, Panel A). She was managed conservatively, and follow-up CT on Day 4 confirmed no enlargement; she was discharged. From Day 5, she gradually became somnolent and developed progressive right-sided weakness. On Day 7, she was transferred to our hospital for further evaluation.

On arrival, her Glasgow coma scale (GCS) score was E3V4M6 (eye opening 3, verbal response 4, and motor response 6), and she exhibited right hemiparesis with Manual Muscle Testing (MMT) grade 3 (movement against gravity but not against resistance). Vital signs were stable (temperature 36.4°C, heart rate 80 beats/min, blood pressure 139/98 mmHg, and SpO₂ 98% on room air). Pupils were equal without anisocoria, and bilateral pupillary light reflexes were intact. CT revealed enlargement of the left ASDH to 15 mm with a 5-mm midline shift (Figure 1, Panel B). Because of progressive neurological deterioration together with increased mass effect on CT, we proceeded to emergency hematoma evacuation.

Her history included gastric and hypopharyngeal cancer, both surgically treated. The hypopharyngeal carcinoma had been treated with radiation, and endoscopic evaluation revealed laryngeal edema, suggesting a risk of airway obstruction during sedation; therefore, general anesthesia was selected for safety.

**Figure 2:**
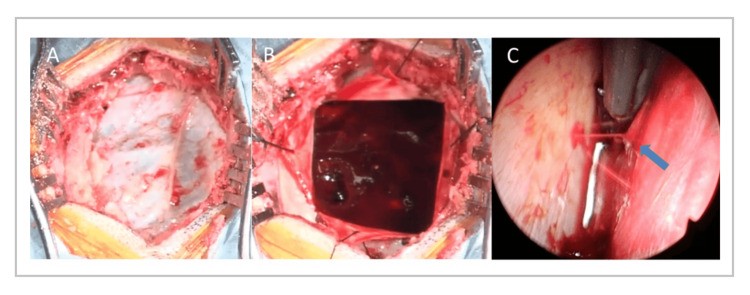
(A) Intraoperative photograph after craniotomy showing preserved dural pulsation, indicating that intracranial pressure was not markedly elevated at the time of exposure. (B) Intraoperative photograph after dural incision showing a dark-red, gelatinous hematoma, consistent with an acute subdural hematoma rather than a chronic subdural hematoma. (C) Endoscopic view after hematoma removal demonstrating an active arterial bleeding point from a cortical artery (arrow), which was treated with coagulation.

Emergency endoscopic-assisted hematoma evacuation was performed on the day of admission. The incision site was selected over the thickest portion of the subcutaneous hematoma, perpendicular to the orbitomeatal line, immediately above the external auditory canal. A 7-cm linear incision was made, and two burr holes were created (Figure 2, Panel A). A craniotomy measuring approximately 4 × 5 cm was created by connecting the burr holes. This size was chosen to allow rapid decompression and safe instrument handling while preserving the option for immediate conversion to a wider exposure if significant brain swelling or uncontrolled bleeding was encountered. A rigid endoscope was used to extend visualization beyond the bony window and to facilitate targeted hemostasis in areas not directly visible through the craniotomy. After dural incision, a dark-red, gelatinous hematoma was observed (Figure 2, Panel B). The superficial clot was removed by suction, and the rigid endoscope was inserted. A bleeding cortical artery was identified and coagulated (Figure 2, Panel C).

**Figure 3:**
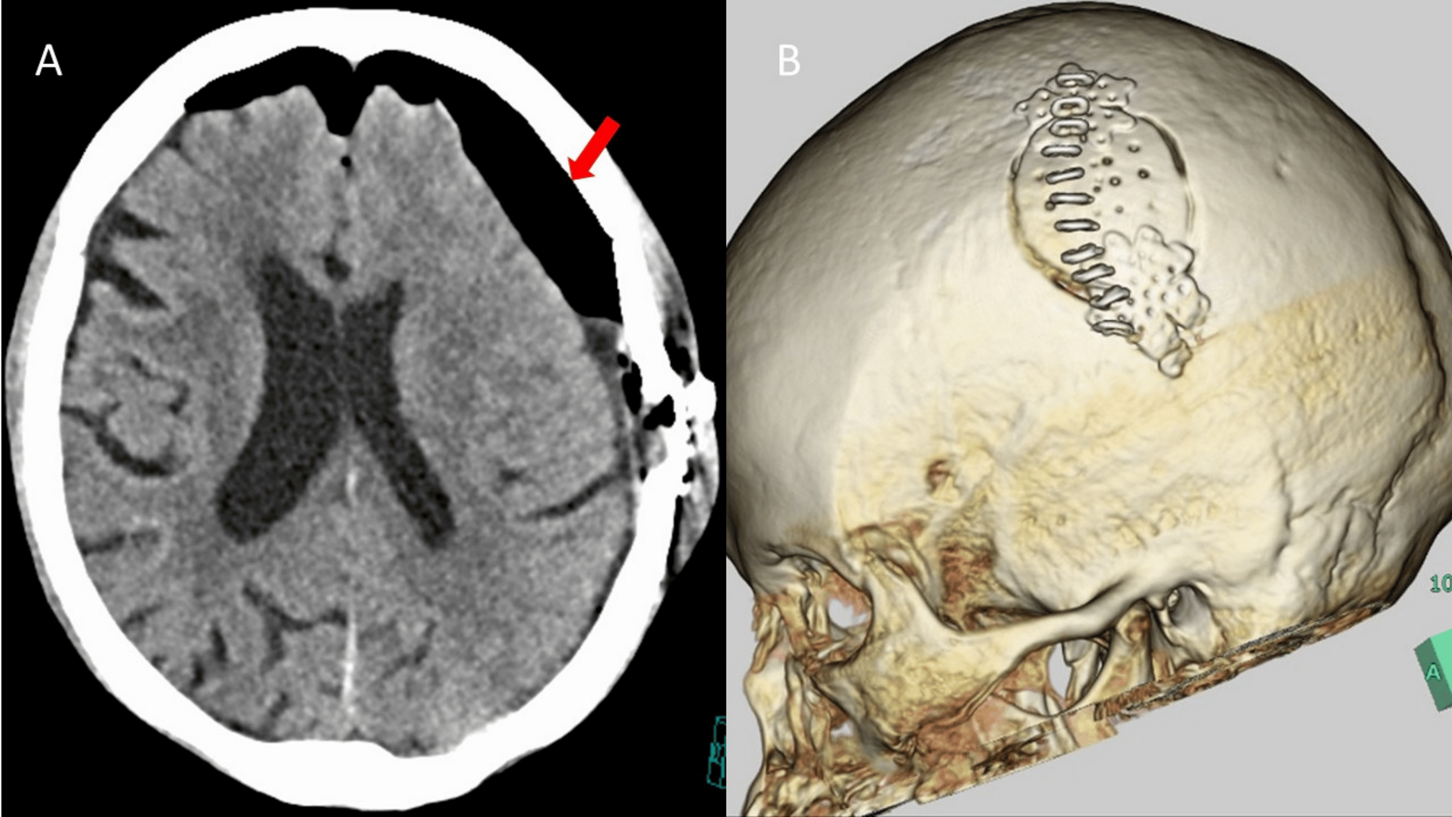
(A) Postoperative head CT obtained immediately after surgery showing complete removal of the hematoma (arrow) and improvement of mass effect. (B) Postoperative 3D CT image demonstrating the 7-cm linear skin incision and the craniotomy site corresponding to the thickest portion of the scalp (subcutaneous) hematoma.

The operative time was 93 minutes, and the estimated blood loss was 100 mL. Postoperative CT confirmed complete hematoma removal (Figure 3, Panels A-B). Postoperatively, her vital signs remained stable (temperature 37.4°C, heart rate 80 beats/min, blood pressure 132/80 mmHg, and SpO₂ 97% on room air), with no anisocoria and intact bilateral pupillary light reflexes. The patient regained full consciousness and was discharged home three weeks later without neurological deficits.

## Discussion

ASDH accounts for 10%-20% of all TBI hospitalizations [1]. Approximately 70% of patients are above 45 years old, and 40% are above 65 years [1]. In elderly patients, brain atrophy increases intracranial space, delaying symptom onset. Such patients often present with an initially lucid interval followed by rapid deterioration (“talk and deteriorate”) [1]. These features complicate early surgical decision-making.

Surgical indications for ASDH have been based on GCS, pupillary findings, and CT findings such as hematoma thickness and midline shift [1]. Traditionally, a large craniotomy or decompressive craniectomy has been performed for severe cases. However, each has disadvantages: craniotomy carries the risk of rebleeding and brain swelling, while decompressive craniectomy may result in wound infection or skull deformity [5]. In a multicenter randomized controlled trial, Hutchinson et al. reported no significant difference in 12-month functional outcomes between craniotomy and decompressive craniectomy groups [5]. Therefore, craniotomy with bone replacement is considered reasonable in suitable cases, particularly when less invasive management is desired.

For elderly or frail patients, smaller craniotomies with endoscopic-assisted evacuation reduce surgical stress and duration while achieving effective decompression [2]. Katsuki et al. defined indications for endoscopic surgery as hematomas compressing the cortex without extensive contusion or severe edema, where decompression is not required [2]. Yokosuka et al. demonstrated that endoscopic-assisted evacuation in elderly patients reduces operative time and blood loss [3]. Iimori et al. further showed that preoperative CTA helps identify bleeding sources and plan the optimal craniotomy site [7].

Endoscopic-assisted evacuation can be performed under either general or local anesthesia [2-4]. General anesthesia provides stable conditions and neuroprotection through hyperventilation and hypothermia [2]. However, in elderly patients with impaired cardiopulmonary reserve, local anesthesia is a safer choice [4]. In this case, due to prior radiation therapy and airway risk, general anesthesia was selected.

We made a 7-cm incision over the thickest hematoma area, allowing for possible conversion to a large craniotomy. While craniotomy provides wider exposure, endoscopic-assisted surgery enables gradual suction of the hematoma and precise hemostasis with minimal brain manipulation. Yokosuka et al. recommended the thickest hematoma site for craniotomy, whereas Hsiao et al. suggested a slightly posterior or superior position to enhance visualization [3,8]. Additional endoscopic-assisted ASDH series have also reported acceptable operative times and clinical outcomes with small craniotomies, as summarized in Table 1, further supporting this technique as a complementary option in carefully selected patients [9-13]. Tanaka et al. noted that the presence of cerebral contusion or intraparenchymal hematoma should be considered an exclusion criterion for endoscopic-assisted evacuation, which was not present in our case [10]. ICP monitoring postoperatively may prevent over-decompression and maintain cerebral perfusion [4]. Recent reports emphasize combining endoscopic-assisted evacuation with ICP-guided postoperative management [4].

**Table 1:** Review of past case series NA: Not addressed; ASDH: Acute subdural hematoma; GCS: Glasgow coma scale.

References	Number of patients	Mean age	Craniotomy size	Approach site	Anesthesia	Mean operative time	Blood loss	Mean clinical outcome	Indications	Contraindications
Katsuki et al. [2]	15	86	2.5 cm	Hematoma thickest	Local	91 min	20cc	mRS5.0	A patient’s comorbid burden, patients less likely to require decompressive craniectomy	An enlarging hematoma
Yokosuka et al. [3]	11	82.6	2.5 cm	Center of hematoma	Local	85.3 min	NA	mRS1.5	Symptomatic, age older than 70 years	Moderate or massive brain contusion/hematoma, an enlarging SDH, and high risk of bleeding
Tanaka et al. [4]	9	82	5.5 cm	Hematoma thickest	General	98.5 min	NA	mRS4.7	Symptomatic	The presence of contusion/hematoma
Hsiao et al. [8]	6	71.2	2 cm	Slightly posterior to the center of the hematoma	General	155.5 min	NA	mRS1.0	Persistent symptoms or episodes of consciousness alteration	Significant brain contusion or intra-parenchymal hematoma, ASDH >10 mm or midline shift > 5 mm, severe brain swelling
Sam et al. [9]	6	77	3 cm	Hematoma thickest	Local	NA	NA	GOS3.8	GCS ≥ 9 with reactive pupils initially, ASDH > 10 mm or midline shift > 5 mm, symptomatic	Coagulopathy, intracerebral hematoma, burst lobes
Hwang et al. [10]	13	78.6	3.5 cm	At the center of the superior temporal line and the coronal suture	Local/general	78.4 min	NA	GOS3.0	Age > 65 years with brain atrophy, decreased consciousness, large hematoma with brain shift, presence of medical morbidities, relatives’ refusal of decompressive surgery	Severe coagulopathy
Kawasaki et al. [11]	17	79.2	3.5 cm	Center of hematoma	Local/general	107.8 min	NA	mRS2.7	Patients’ comorbid burden, patients less likely required decompressive craniectomy, ASDH > 15 mm	Uncorrectable severe coagulopathy
Khattar et al. [12]	3	44.3	2 cm	Superior to the stephanion	NA	206 min	NA	mRS1.3	Hematoma thickness ≥ 10 mm, hematoma volume ≥ 30 cm³, cerebral edema with midline shift ≥ 5 mm, progressive neurological decline	GCS ＜ 13 and were rapidly deteriorating
Ichimura et al. [13]	5	87.4	3 cm	Hematoma thickest	Local/general	NA	NA	mRS4.2	Symptomatic inability to use a large craniotomy because of poor general condition or absence of an anesthesiologist	Moderate or massive brain contusion or edema
Present case	1	76	4 × 5 cm (max 5 cm)	Thickest subcutaneous hematoma; above external auditory canal; perpendicular to orbitomeatal line	General anesthesia (laryngeal edema; airway risk with sedation)	93 min	100 mL	Neurologically intact at discharge (3 weeks)	Meets Katsuki: less likely to require decompressive craniectomy; endoscope for extended visualization and targeted hemostasis	No contusion/intraparenchymal hematoma (Tanaka exclusion not met)
Benchmarks (from included studies; total N = 85)			2–5.5 cm (median 3)			78.4–206 min (median 98.5)	20–20 mL (median 20)	mRS mean 1–5 (median 2.7); scales varied		

This case illustrates the feasibility of endoscopic-assisted evacuation under general anesthesia in a high-risk, elderly patient with a history of radiation therapy. The operation was completed safely, and the patient recovered without deficits. When airway safety is ensured, endoscopic-assisted evacuation can be a valuable, less invasive alternative to craniotomy. Further case accumulation will refine patient selection, anesthesia choice, and craniotomy placement.

Endoscopic-assisted hematoma evacuation is less invasive and requires a shorter operative time than conventional craniotomy. When carefully selected, it offers a safe and effective treatment option for ASDH, particularly in elderly or high-risk patients.

## Conclusions

Endoscopic-assisted hematoma evacuation may be a safe and effective alternative to conventional craniotomy for ASDH in carefully selected patients, particularly elderly or high-risk individuals. In this case, the technique enabled adequate hematoma evacuation and targeted hemostasis through a limited exposure with minimal brain manipulation. However, operative time and technical difficulty can vary, and this approach is not universally faster or applicable, especially in the presence of marked brain swelling.

Previous reports have described the feasibility of endoscopic-assisted evacuation under both local and general anesthesia. Consistent with these reports, the present case suggests that even patients with airway concerns may safely undergo endoscopic-assisted evacuation under general anesthesia when appropriate airway management is ensured. As this is a single case, these findings should not be overgeneralized. As the population ages, further case accumulation and multicenter studies are needed to better define indications and contraindications, refine techniques, and evaluate long-term outcomes.
